# Determination of the phylogenetic origins of the Árpád Dynasty based on Y chromosome sequencing of Béla the Third

**DOI:** 10.1038/s41431-020-0683-z

**Published:** 2020-07-07

**Authors:** Péter L. Nagy, Judit Olasz, Endre Neparáczki, Nicholas Rouse, Karan Kapuria, Samantha Cano, Huijie Chen, Julie Di Cristofaro, Goran Runfeldt, Natalia Ekomasova, Zoltán Maróti, János Jeney, Sergey Litvinov, Murat Dzhaubermezov, Lilya Gabidullina, Zoltán Szentirmay, György Szabados, Dragana Zgonjanin, Jacques Chiaroni, Doron M. Behar, Elza Khusnutdinova, Peter A. Underhill, Miklós Kásler

**Affiliations:** 1 Department of Pathology, Laboratory of Personalized Genomic Medicine, Columbia University, New York, NY USA; 2 National Institute of Oncology, Budapest, Hungary; 3 Department of Archaeogenetics, Institute of Hungarian Research, Budapest, Hungary; 4 Department of Genetics, University of Szeged, Szeged, Hungary; 5 MNG Laboratories LLC, Atlanta, GA USA; 6 Aix Marseille Université, CNRS, EFS, ADES, “Biologie des Groupes Sanguins”, Marseille, France; 7 Gene by Gene, Houston, TX USA; 8 Department of Genetics and Fundamental Medicine, Bashkir State University, Ufa, Russia; 9 Institute of Biochemistry and Genetics - Subdivision of the Ufa Federal Research Centre of Russian Academy of Sciences, Ufa, Russia; 10 Department of Pediatrics and Pediatric Health Center, University of Szeged, Szeged, Hungary; 11 King St. Stephen Museum, Székesfehérvár, Hungary; 12 Gyula Siklósi Research Centre for Urban History Székesfehérvár, Székesfehérvár, Hungary; 13 Gyula László Department and Archive, Institute of Hungarian Research, Budapest, Hungary; 14 Institute of Forensic Medicine, Clinical Center of Vojvodina, Novi Sad, Serbia; 15 Faculty of Medicine, University of Novi Sad, Novi Sad, Serbia; 16 Estonian Biocentre, Institute of Genomics, University of Tartu, Tartu, Estonia; 17 Department of Genetics, Stanford University, Stanford, CA USA; 18 Present Address: Praxis Genomics LLC, Atlanta, GA USA; 19 Present Address: Boston’s Children’s Hospital, Boston, MA USA

**Keywords:** Genome evolution, Data mining

## Abstract

We set out to identify the origins of the Árpád Dynasty based on genome sequencing of DNA derived from the skeletal remains of Hungarian King Béla III (1172–1196) and eight additional individuals (six males, two females) originally interred at the Royal Basilica of Székesfehérvár. Y-chromosome analysis established that two individuals, Béla III and HU52 assign to haplogroups R-Z2125 whose distribution centres near South Central Asia with subsidiary expansions in the regions of modern Iran, the Volga Ural region and the Caucasus. Out of a cohort of 4340 individuals from these geographic areas, we acquired whole-genome data from 208 individuals derived for the R-Z2123 haplogroup. From these data we have established that the closest living kin of the Árpád Dynasty are R-SUR51 derived modern day Bashkirs predominantly from the Burzyansky and Abzelilovsky districts of Bashkortostan in the Russian Federation. Our analysis also reveals the existence of SNPs defining a novel Árpád Dynasty specific haplogroup R-ARP. Framed within the context of a high resolution R-Z2123 phylogeny, the ancestry of the first Hungarian royal dynasty traces to the region centering near Northern Afghanistan about 4500 years ago and identifies the Bashkirs as their closest kin, with a separation date between the two populations at the beginning of the first millennium CE.

## Introduction

The Árpád Dynasty (ca. 850–1301 CE) established the Hungarian state in the Carpathian Basin and played a formative role in Eastern European history. The Dynasty was founded by Prince Álmos (ca. 820 CE–ca. 894 CE), but in the modern historiography got its name from his son, Prince Árpád (ca. 845 CE–ca. 907 CE) who ruled between ca. 894 CE and ca. 907 CE and lead the Hungarians into the Carpathian basin between 862 CE and 896 CE [1]. Although partial mitochondrial and Y-chromosome analyses of remains from cemeteries of conquering Hungarian nobility have been performed, the ethnic origins of the Árpád Dynasty are subject to scientific debate in the absence of genetic evidence [2–6]. Historical sources indicate that ten Árpáds, eight kings, and two princes, were laid to rest in the provostry church of the Virgin Mary, commonly known as the Royal Basilica of Székesfehérvár, before the Turkish occupation of that city in 1543 [7–9]. During the following centuries of war and neglect, the Basilica was destroyed, and the only royal graves left undisturbed were those of King Béla III (ca. 1148–1196) and his first spouse, Anna of Antioch (ca. 1150–1184/85). The royal remains were discovered in 1848 by the leading archaeologist and historian of the time, János Érdy, a member of the Hungarian Academy and curator of the National Museum in Budapest [10]. The royal regalia unearthed with the remains of Béla III and Anna of Antioch are in the possession of the National Museum of Hungary in Budapest. The remains were reinterred first in the undercroft of Matthias Church of Buda in 1862 and later in the Holy Trinity Chapel of the church in 1898. Additional remains from the site of the Royal Basilica of Székesfehérvár were excavated in 1862 and 1874 and eight of these were placed in the Matthias Church in 1900 [11].

Y-chromosomal Short Tandem Repeat (STR) analysis of King Béla III and six other unidentified male skeletons indicated that in addition to Béla III, only one of the remains, marked HU52, belongs to the Árpád Dynasty [7].

Developments in next-generation sequencing (NGS) during the past decade have made it possible to economically sequence the mitochondrial and Y chromosomes in large number of individuals [12]. Two pivotal studies [13, 14] sequenced 456 and 1244 globally diverse samples, respectively, leading to the identification of over 65,000 Y-chromosome SNPs. Since these papers were published, the number of known Y-chromosomal SNPs has tripled allowing high-resolution analysis of patrilineal relationships [15]. We undertook NGS of the DNA derived from the remains studied by Olasz et al. [7] and determined the phylogenetic origins and closest kinship of the Árpád Dynasty based on shared Y chromosome haplogroup derivation in the context of 40 Eurasian populations.

## Materials and methods

### Ancient samples

The ancient remains examined in this study are under the legal guardianship of the Hungarian Catholic Church. Péter Erdő, Archbishop of Esztergom-Budapest, provided written consent to perform genetic analysis on the remains that was sanctioned by the Hungarian Ministry of Human resources (EMMI) under project number 26090/2019/MINKABINET.

Sample collection was performed at the National Institute of Oncology in Budapest, Hungary and DNA extraction of the nine ancient skeletal remains was performed in the laboratory of Susanna Hummel at the Department of Historical Anthropology and Human Ecology of the Johann-Friedrich-Blumenbach Institute for Zoology and Anthropology [7]. Next-generation sequencing (NGS) and primary data analysis were performed at Praxis Genomics LLC (Atlanta, GA) and the results were verified by reanalysis of all data at the Department of Archaeogenetics, Institute of Hungarian Research, Budapest, Hungary.

Further technical detail about the NGS process and data analysis is provided in the “Library preparation and NGS” and “Data analysis” sections. All handling of DNA samples were under strict observance of CLIA and CAP guidelines.

### Modern samples

**Fig. 1:**
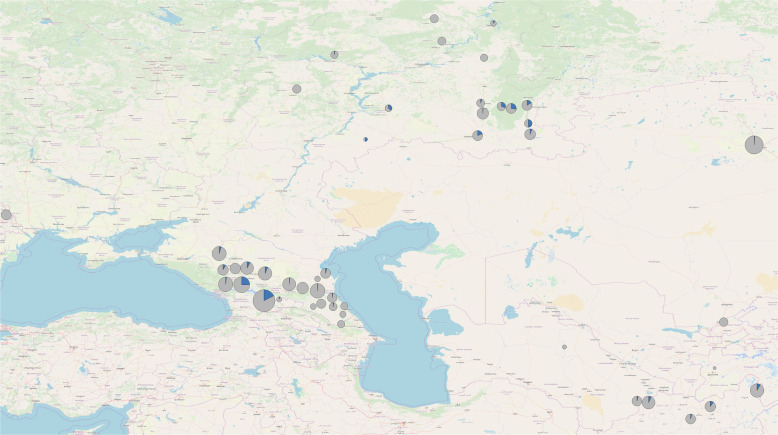
Prevalence of the R-Z2123 haplogroup (R1a1a1b2a2a1) based on 4340 modern samples from 40 different Eurasian populations (Table S1). The map was created using QGIS 3.8. Zanzibar [43]; circle size correlates with the size of the population tested; Blue slices in gray background represent the percentage of the R-Z2123 haplogroup in these populations.

4340 modern samples from 40 different populations were genotyped for the Z2125 and Z2123 SNPs using Sanger sequencing (Fig. 1, Table S1). 206 samples belonging to the R-Z2123 haplogroup from 20 populations were paired-end sequenced (2 × 151 bp) using Novaseq 6000 (Illumina) (Fig. 1, Table S1). Use of these samples for this study was approved by the Ethics Committee of the Ufa Federal research Centre of the Russian Academy of Sciences; document number: MKI-F/321-1/2019. Further technical detail is provided in the “Library preparation and NGS” section. All subjects were voluntary participants and gave written consent to use their samples for genetic analysis. In addition, two published Y-chromosome sequencing datasets corresponding to an Iraqi and an “Iraqi Jew”, were downloaded from the European Nucleotide Archive (http://www.ebi.ac.uk/ena) under the accession number PRJEB21310 and were used in the analysis [16].

### Library preparation and next-generation sequencing

Modern DNA samples were sonicated using Covaris S220 Ultrasonicator (Woburn, MA) to yield fragments with a median fragment length of 300 bp according to the manufacturer’s recommendations. For ancient DNA samples, no sonication was necessary. Low-molecular weight DNA (<300 bp) enrichment from all samples was performed using AMPure XP beads (Beckman Coulter, Indianapolis, IN). Library preparation was performed using the TruSeq Nano DNA LT kit (Illumina, San Diego, CA) according to the manufacturer’s recommendations. Library size and quality was confirmed with Fragment Analyzer (Advanced Analytical, Santa Clara, CA) and quantitation was done using qPCR (S1000/CFX96 Real Time System; Biorad, Hercules, CA) Paired-end sequencing (2 × 125 bp; and 2 × 151 bp) was performed on HiSeq 2500 and NovaSeq 6000 Systems (Illumina) following the manufacturer’s recommendations.

### Data analysis

Initial analysis of both ancient and modern datasets was performed at Praxis Genomics LLC (Atlanta, GA). Demultiplexing of NGS runs was performed in BaseSpace (Illumina, San Diego) [17]. Adapter and quality trimming was performed on the ancient samples by Trim Galore (Babraham Bioinformatics) [18]. Reads with length under 40 bp after quality and adapter trimming were removed and only properly paired read data were retained and aligned to the GRCh38 reference by Dragen v.3.2.5 (Illumina) using default settings. Variant calling was also performed using Dragen. The Integrative Genome Viewer [19, 20] was used for visual confirmation of calls made.

The conclusions of the Dragen alignment and variant calling were corroborated by reanalysis of the ancient datasets at the Department of Archaeogenetics, Institute of Hungarian Research, Budapest, Hungary. The ancient datasets were aligned to both GRCh37 (hs37d5) and GRCh38 using Burrow-Wheels-Aligner (v 0.7.17) using the MEM command with reseeding disabled. PICARD tools were used to mark duplicates and only properly paired primary alignments with ≥90% identity to reference were considered in all downstream analyses.

Ancient DNA damage patterns were assessed using MapDamage 2.0 [21] and read quality scores were modified with the Rescale option to account for post-mortem damage. Sex determination was performed according to the method described in Skoglund et al. [22]. Mitochondrial genome contamination was estimated using Schmutzi algorithm [23] (Table 1). Contamination for the male samples was assessed by the ANGSD X chromosome contamination method [24].

**Table 1:** Summary of statistical analysis of NGS of ancient samples.

Sample identifier	HU3B	HU3G	HU4H	HU52	HU53	HU54	HU55	HU109	HUAA
Site of origin	MT	TA	MT	TA	ST	CO	CO	CO	CO
Extraction ID	EX5	EX3	EX3	EX3	EX4	EX3	EX5	EX4	EX5
Total reads mapped to GRCh37 (million)	203.1	278.6	188.7	164.2	29.0	33.2	49.2	116.7	7.1
Duplicate marked read pairs (million)	3.9	8.2	3.9	26.2	0.5	0.4	0.6	8.7	1.2
Number of unique reads mapped to GRCh37 (million)	195.4	262.1	180.8	111.8	28.0	32.3	48.0	99.4	4.7
Avg. coverage over genome (fold)	7.02	9.62	6.52	4.30	1.04	1.21	1.76	3.80	0.17
Endogenous DNA content	5.24%	50.65%	39.22%	4.40%	1.10%	2.79%	4.28%	20.13%	0.19%
Avg. X-chromosome coverage	3.81	5.36	3.63	2.32	0.59	0.66	0.95	4.01	0.18
Avg. Y-chromosome coverage	4.33	5.67	3.09	2.18	0.44	0.55	0.80	0.06	0.00
Avg. mitochondrial coverage (fold)	453	721	497	304	356	121	101	274	23
Mitochondrial bps covered >10-fold	100.00%	100.00%	100.00%	100.00%	100.00%	99.90%	99.90%	100.00%	94.20%
Number of mitochondrial bases not covered	0	0	0	0	0	0	0	0	19
Estimated mitochondrial contamination with Schmutzi	1.00%	1.00%	1.00%	1.00%	5.00%	1.00%	2.00%	1.00%	2.00%
Estimated X contamination with ANGSD	1.21%	1.01%	0.30%	1.83%	11.94%	1.30%	1.34%	–	–

The origin of the bone material, which was used for DNA extraction: MT – metatarsus; TA – tarsus; ST – sternum; CO – costa. Total reads mapped to GRCh37 (million) are properly paired primary alignments with ≥90% identity to reference genome. Additional detail is provided Table S3.

Mitochondrial Haplogroup determination was performed using HaploGrep [25] (Tables 2, and S2). Y chromosome haplogroup determination was initially performed using Yleaf [26]. SNPs described in FamilyTreeDNA Y-DNA Haplotree and ISOGG SNP databases [27, 28] were used to further interrogate genomic datasets using bam-readcount program [29]. Bam readcount provides depth of coverage, nucleotide composition, and read quality at the specified chromosomal positions. This approach allowed us to further derive the Y-leaf generated haplogroups, and to identify a novel haplogroup defining the Árpád Dynasty (R-ARP). We used Clustal Omega Version 1.2.4 [30] to reconstruct the R-Z2123 haplogroup phylogeny and calculate coalescence times between derived haplogroups. Only SNPs that fall within the ~10 Mb region of the Y-chromosome described by Poznik et al. [13] were used in the calculation of coalescence times. Drawing of the phylogenetic tree was performed using FigTree v1.4.4 [31].

**Table 2:** Ancient samples’ Y and mitochondrial haplogroup assignments.

Sample identifier	HU3B	HUAA	HU52	HU3G	HU4H	HU53	HU54	HU55	HU109
Sample description	Béla III, Southern aisle, marble coffin; Burial: 1196	Anna of Antioch; Adjacent to Béla III in southern aisle; Burial: 1184	Adjacent to Béla III in southern aisle; Burial: presumably prior to Béla III	Northern aisle of church, stone lined grave	Northern aisle, stone lined grave	Inside the church, site not specified	Inside church; site not specified	Inside the church, site not specified	Inside church; site not specified
Y chromosome haplogroup (Familytree)	R-SUR51^a^	woman	R-SUR51^a^	J-ZS7626^a^	R-PF6658	E-BY4992	R-YP1626^a^	R-BY41605	woman
Y chromosome haplogroup (ISOGG, Yleaf)	R1a1a1b2a2a1c3	woman	R1a1a1b2a2a1	J1a2b1b2c1a	R1b1a1b1a1a2b	E1b1b1a1b1a	R1a1a1b1a2b3a4a	R1b1a1b1a1a1c2	woman
Mitochondrial Hg (HaploGrep)	H1b	H7b1	T2b2b1	U5b2c	U4a	H1c1	U4a2b	J1c3a	H46

Y chromosomal haplogroup defining SNPs are listed in Tables S4 and S5.

^a^Indicates the FamilyTree based haplogroups beyond the ISOGG assignments. Mitochondrial haplogroup defining SNPs are listed in Table S2.

The BAM files of the mitochondrial and Y chromosome sequences for all samples presented in this paper are available at https://www.ncbi.nlm.nih.gov/bioproject/PRJNA490697: Determination of the phylogenetic origins of the Árpád Dynasty based on Y-chromosome sequencing of Béla the Third.

## Results

### Next-generation sequencing data

We have performed NGS on nine ancient DNA samples. Unique reads per sample, endogenous DNA content, X and Y chromosome, as well as autosomal coverage varied widely between samples (Tables 1 and S3). X and Y chromosome coverage was calculated for the entire length of the chromsomes. Y and X chromosome read ratios allowed unequivocal assignment of chromosomal sex in all samples (Table S3b) [22]. Mitochondrial genomes for all individuals had >100-fold average coverage except sample HUAA (Anna of Antioch), for whom we only obtained 23-fold average coverage. The estimated X contamination based on the ANGSD X chromosome contamination assessment tool varied from 0.3 to 1.34% [24], except for one sample, HU53, in which it was considerably higher (11.94%). The estimated mitochondrial contamination determined by Schmutzi varied from 1 to 2% for most samples, but was at 5% in HU53 (Table 1). The overall amount and quality of the data allowed precise derivation of mitochondrial and Y chromosomal haplogroups in all samples studied (Table 2, Figures S1, S2 and Tables S2, S4, 5).

### Y chromosome haplogroup derivation for ancient samples

**Fig. 2:**
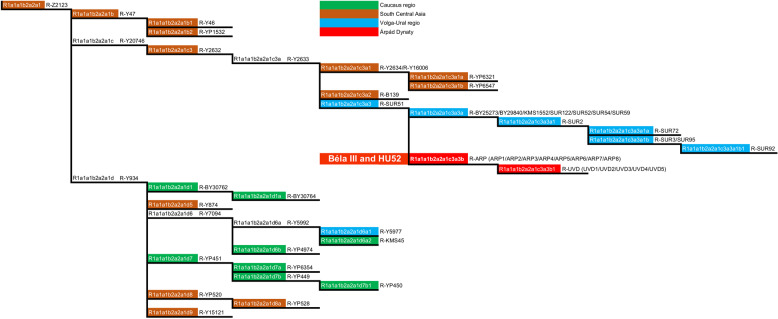
Haplogroup derivation of Árpád Dynasty members Béla III and HU52 in the context of 208 modern R-Z2123 haplogroup (R1a1a1b2a2a1) individuals. The branch architecture presented is based on ISOGG and FamilyTreeDNA Y chromosome haplotrees. Background coloring reflects the predominant geographical origins of the individuals used in this study derived for the respective haplogroups with the exception for the red color, that denotes the Árpád Dynasty haplogroup.

The Yleaf software outputs Y-chromosome haplogroups for male samples in the format of ISOGG (Tables 2 and S4). In all cases, haplogroups output by Yleaf could be further derived using manual asessment of data based on Family Tree haplogroup and SNP information. For this reason, for each individual haplogroup derivation, the Family Tree based haplogroup is provided along with the ISOGG based haplogroup assignment (Fig. 2, Tables 2 and S5).

The HU53 sample was derived for the E-BY4992 (E1b1b1a1b1a) haplogroup based on brachpoint SNPs BY4991 and BY4999 that could be ascertained. This haplogroup is frequent in the southern Balkans, especially in Greece [32] and was also detected Hungarian conquerors and Avars [6].

The HU3G sample was derived for haplogroup J-ZS7626 (J1a2b1b2c1a2b) based on the presence of 6 out of 25 haplogroup defining SNPs. This haplogroup radiates across eastern Africa and the southern tip of the Arabian Peninsula, but is also seen among inhabitants of the western shores of the Caspian Sea among Tabasaran, Kumyk, Avar, and Lezgin populations [14, 33].

The most common haplogroup among the remains tested was the Eurasian R1 haplogroup that bifurcates into western (R1b) and eastern (R1a) branches [34, 35].

Sample HU55 is derived for R-BY41605 (R1b1a1b1a1a1c2). The R-U106 haplogroup upstream of R-BY41605 is most common in western Europe [34], and quite frequent among Hungarian conquerors [6].

Sample HU4H is derived for R-BY3642 past R-PF6658 (R1b1a1b1a1a2b). Consistent with this result the R-BY3636; R-BY3630; R-BY3851; R-FGC30121; R-BY42688; R-Y16335 haplogroup defining SNPs downstream of R-PF6658 are not derived and thus these branches can be excluded. The geographic distribution of R1b-U152, that is upstream of R-BY3642 is mainly restricted to the Alpine area of Italy and Corsica [34].

Samples HU3B, HU52, and HU54 belong to haplogroup R1a. HU54 is derived for R-YP1626 (R1a1a1b1a2b3a4a2c) which is most common in southwest Russia and Ukraine, Belarus and eastern Poland [35].

**Fig. 3:**
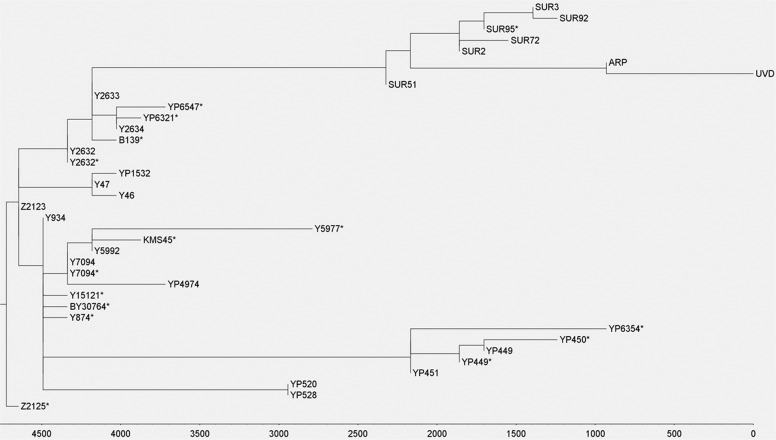
Phylogenetic tree of haplogroup R-Z2123 (R1a1a1b2a2a1). Phylogenetic tree based on 208 high coverage modern Y-chromosome datasets and two ancient Y-chromosome sequences belonging to Árpád Dynasty members Béla III and HU52 generated using Clustal Omega (v.1.4.4.). Sub-clade names are indicated on the banches. Estimated coalescence times are indicated at the button of the figure as “years before present”. All SNPs used for the preparation of this figure fall within the male specific region of Y chromosome and are listed in Table S8.

The remains belonging to Béla III (HU3B) and HU52 are derived for R-Z2125 (R1a1a1b2a2a). The R-Z2125 haplogroup is common in northeastern Afghanistan, Tajikistan, Kyrgyzstan, and southern Kazakhstan and to a lesser extent in the Volga Ural region, the Caucasus and Iran (Fig. 1) [35]. We could further derive the Árpád Dynasty lineage based on SNPs defining R-Y2632, R-Y20746, R-Y2633, and 16 SNPs associated with haplogroup R-SUR51 (Figs. 2 and 3, Table S5) [13, 14, 16, 36, 37].

### High resolution characterization of the R-Z2123 haplogroup

4340 samples from over 40 populations from the Volga-Ural region, the Caucasus, Central and South Central Asia were genotyped for R-Z2125 and/or R-Z2123 (Fig. 1, and Table S1). 400 plus samples were shown to be derived for R-Z2125 and 320 samples were derived for the R-Z2123 haplogroup. 206 representative R-Z2123 samples were chosen for whole-genome sequencing to provide high-resolution characterization of this haplogroup (Figs. 2 and 3). Two previously published high resolution Y chromosome sequencing datasets of an Iraqi and an Iraqi Jew were also included in the analysis along with the two Árpád Dynasty members, Béla III (HU3B) and HU52 [16] (Table S6). From these 210 individuals, five Afghans and one Chechen could not be derived beyond R-Z2123* (R1a1a1b2a2a1). Twelve individuals, all from Afghanistan, are derived for R-Y47 (R1a1a1b2a2a1b). 58 individuals, including 48 Bashkirs predominantly from the Burzyansky and Abzelilovsky districts of Bashkortostan, 4 Afghans [38], 2 Árpád Dynasty members, an individual from modern day Serbia [7, 39], a Punjabi from Lahore (HG03636) [13], an Iraqi Jew (GRC14414377 a.k.a 16198) [14, 16, 36] and an Iraqi (GRC15570738) [16] were derived for R-Y2632 (R1a1a1b2a2a1c3). While the Iraqi individual (GRC15570738) could not be derived past R-Y2632* (R1a1a1b2a2a1c3), the other 57 samples are all derived for R-Y2633 (R1a1a1b2a2a1c3a). Post Y2633, these individuals form three different haplogroups defined by SNPs B139 (present only in the Iraqi Jew), Y2634 and Y16006 (present in the Punjabi from Lahore (HG03636) and 4 Afghans, and SUR51 present in the 48 Bashkirs, the individual from modern day Serbia and the two Árpád Dynasty members, Béla III (HU3B) and HU52. The R-SUR51 haplogroup bifurcates further after 17 shared SNPs (Table S7). The Bashkirs have the additional six SNPs associated with R-SUR51, while the individual from modern day Serbia and the two Árpád Dynasty members lack these, but are derived for a novel haplogroup, R-ARP, defined by nine shared SNPs. The individual from modern day Serbia has nine additional private SNPs, absent from the ancient samples, that define a novel haplogroup R-UVD (**U**j**v**i**d**ék; the Hungarian name of modern day Novi Sad). 38 Bashkirs are further derived for R-SUR2 and its subhaplogroups R-SUR72 and R-SUR3/SUR95 while 7 have not been derived past R-SUR51*.

The other 134 individuals, including all 89 samples from the Caucasus, 30 Bashkirs predominantly from the Arhangelsky region and 15 Afghans are derived for Y934 and its subhaplogroups (Figs. 2 and 3, Table S6).

We generated a phylogenetic tree of the 208 modern samples and the two Árpád Dynasty members described above using the Clustal Omega software (v.1.2.4) [30] to assess evolutionary relationships and time to most recent common ancestor. Only SNPs that fall within the ~10 Mb region of the Y-chromosome described by Poznik et al. [13] were used in the calculation of coalescence times (Table S8). Using the appearance of R-Y2633 haplogroup 4100 years ago as an anchor based on prior work [16], the data indicates that the individual from modern day Serbia and the two Árpád Dynasty members, Béla III (HU3B) and HU52 separated from the Bashkirs about 2000 years ago, while the four main branches of the R-2123 haplogroup represented in this cohort, R-Z2123*, R-Y47, R-Y2632, R-Y934 arose ~4500 years ago.

## Discussion

Our objective was to identify and characterize SNPs defining the Árpád Dynasty and establish their phylogenetic origin.

Y chromosome haplogroup analysis of the seven ancient male skeletons previously Y-STR genotyped by Olasz et al. [7] confirms that only one, HU52, shares the Y chromosome haplogroup R-ARP with King Béla III confirming the hypothesis that this individual belongs to the House of Árpád. The other individuals are of diverse origins (J-ZS7626, E-BY4992, R-BY41605, R-BY3642, and R-Y2608) and are not related to the royals or to each other along paternal lineages. Mitochondrial haplogroup analysis revealed different haplogroups for all samples indicating no shared maternal lineage (Tables 2 and S2). The fact that neither Anna of Antioch nor Béla III share mitochondrial haplogroup with HU52 excludes the possibility that HU52 is their son, or Béla III’s brother. He could be Béla III’s father King Géza II (lived 1130–1162), or grandfather Béla II (1110–1141) or a more distant ancestor.

Identification of two members of the Árpád Dynasty provided us with the tools to address the phylogenetic origins of this dynasty. Based on 17 shared R-SUR51 SNPs, we established that Bashkirs, predominantly from the Burzyansky and Abzelilovsky districts, are the closest kin of the Árpád Dynasty from among the populations examined (Table S7). We further derived the Árpád Dynasty Y-chromosome haplogroup to R-ARP (**Árp**ád) (proposed to be equivalent to R1a1a1b2a2a1c3a3b following ISOGG naming conventions) based on nine shared SNPs between them and an individual from modern day Serbia.

It was proposed by multiple authors that one new Y chromosome SNP gets introduced into the germline every 100–150 years [16]. Based on this calibration, Behar et al. (2017) estimated that the Iraqi and the Iraqi Jew lineages diverged ~4100 years ago [16] coinciding with the appearance of the Y2633 SNP in the latter individual. Using this date as an anchor, the coalescence estimation using the Clustal Omega software suggests that the Z2123* starburst (appearance of R-YP3920, R-YP4907, R-Y47, R-Y934, and R-Y2632) occurred >4500 years ago (Fig. 3). This likely occurred in the region centered on northern Afghanistan since all Z2123* individuals of our cohort, as well as individuals belonging to the early branches of the R-Y47 (R-46), R-Y934 (R-Y874*, R-Y15121*, R-YP520*) and the R-Y2632 haplogroups (R-Y2634 and R-Y16006; R-YP6321*; R-YP6547*) are seen predominantly in Afghan individuals [35]. The more derived haplogroups of R-Y2632, (R-SUR51) and R-Y934 (R-YP451 and R-Y5977) are to be found in the Volga Ural and the Caucasus regions, respectively, suggesting a founder effect and a relatively recent population expansion at their current locations.

Our analysis indicates that the ancestral lineage of modern Bashkirs separated from the lineage of the Árpáds about 2000 years ago marked by the appearance of the R-ARP haplogroup. Appearance of the R-UVD haplogroup present in the individual from modern day Serbia is estimated to have occurred about 900 years ago consistent with the burial date (1196) of King Béla III. Of course these numbers are estimates and hinge significantly on the SNPs that are accepted currently as legitimate to be used in such calculations (Table S8). Out of a total of nine ARP SNPs we identified, only eight fell into the region recommended, while of the nine UVD SNPs only 6 could be included in our analysis. In our opinion the region of interest from which SNPs could and should be taken into account for creating phylogenetic trees should be revised based on the better reference sequences available for the Y-chromosome.

The phylogenetic origins of the Hungarians who occupied the Carpathian basin has been much contested [40]. Based on linguistic arguments it was proposed that they represented a predominantly Finno-Ugric speaking population while the oral and written tradition of the Árpád dynasty suggests a relationship with the Huns. Based on the genetic analysis of two members of the Árpád Dynasty, it appears that they derived from a lineage (R-Z2125) that is currently predominantly present among ethnic groups (Pashtun, Tadjik, Turkmen, Uzbek, and Bashkir) speaking Iranian or Turkic languages. However, their closest kin, the Bashkirs live in close proximity with Finno-Ugric speaking populations with the N-B539 haplogroup. A recent study shows that this haplogroup is also found in modern Hungarians [41]. Intriguingly, the most recent separation of the N-B539 derived lineages found in Hungarians and Bashkirs is estimated to have occurred ~2000 years before present [42]. This would suggest that a group of people consisting of a Turkic (R-SUR51) component and a Finno-Ugric (N-B539) component left the Volga Ural region about 2000 years ago and started a migration that eventually culminated in settlement in the Carpathian Basin. Higher resolution studies of the prevalence of the N-B539, R-SUR51, and R-ARP haplogroups in the Carpathian basin are needed to test this hypothesis, both in the current populations, as well as in the remains from cemeteries from the period of the Hungarian invasion. Targeted sampling of the regions of Levédia and Etelköz, proposed earlier living areas of Hungarians north of the Black Sea between the river Don (or Dnieper) and the Eastern Carpathians, could provide further data to determine the precise timing of the Hungarian migrations prior to their entry into the Carpathian Basin. The rarity of the Y-chromosome lineage of the Árpád Dynasty will allow a very detailed and accurate mapping of Hungarian prehistory and identification of additional descendents of the dynasty, which has been a goal of scholars interested in the subject for centuries.

## Supplementary information

Supplementary information captions

S1 Figure

S2 Figure

S1 Table

S2 Table

S3 Table

S4 Table

S5 Table

S6 Table

S7 Table

S8 Table

## Data Availability

The BAM files of the mitochondrial and Y-chromosome sequences for all samples presented in this paper are available at https://www.ncbi.nlm.nih.gov/bioproject/PRJNA490697: Determination of the phylogenetic origins of the Árpád Dynasty based on Y-chromosome sequencing of Béla the Third.
