# Supplementary material for: Determination of the phylogenetic origins of the Árpád Dynasty based on Y chromosome sequencing of Béla the Third
Source: Eur J Hum Genet. 2020 Jul 7;29(1):164–72. doi: 10.1038/s41431-020-0683-z (PMC7809292; doi:10.1038/s41431-020-0683-z)
Supplement: Supplementary file 1 — Supplementary information captions [file 41431_2020_683_MOESM1_ESM.docx]

# Supporting Information Captions

# Supplementary Figures

# S1 Figure. Mitochondrial haplogroup derivation of the 9 adult skeletal remains from the Basilica of Székesfehérvár

# S2 Figure. Y-chromosome haplogroup derivation of the 7 male skeletal remains from the Basilica of Székesfehérvár

# Supplementary Tables

**S1 Table. Haplogroup assignments of 4340 modern samples from 54 different collection sites representing 40 different populations.** The Z2125 and Z2123 SNPs were assessed using Sanger sequencing. Whole genome sequencing was performed on 206 Z2123 derived samples to determine their haplogroup derivation. Y chromosome capture NGS data for two samples (an Iraqi and an Iraqi Jew) were downloaded from the European Nucleotide Archive (http://www.ebi.ac.uk/ena) (16).

**S2 Table. List of SNPs probed against rCRS to derive mitochondrial haplogroups using HaploGrep (25).** Common indels (hotspots) excluded from analysis: 309.1C(C), 315.1C, 523-524del (or 522-523del), 3106del, 16182C, 16183C, 16193.1C(C), 16519C. The table below uses the nomenclature used by HaploGrep. In HGVS description, SNP 263G for example would be NC_012920.1:m.263A>G, where G represents the altered base in comparison with the reference sequence used.

**S3 Table. Quality assessment of ancient DNA NGS data. S3a Table.** Alignment and coverage statistics based on GRCh37-alignment performed using the pipeline of the Department of Archaeogenetics, Institute of Hungarian Research. **S3b Table.** Sex determination result summary based on methodology described in Skoglund et al (22). **S3c Table.** Ancient DNA damage pattern assessment with MapDamage 2.0 (21).

**S4 Table. Ancient samples' Y chromosomal haplogroup determination with Yleaf. S4a Table.** Summary of haplogroup derivation for all ancient samples. **Table S4b.** Call stats for SNPs used by Yleaf to derive Y chromosome haplogroup for sample HU3B. **Table S4c.** Call stats for SNPs used by Yleaf to derive Y chromosome haplogroup for sample HU3G. **Table S4d.** Call stats for SNPs used by Yleaf to derive Y chromosome haplogroup for sample HU4H. **Table S4e.** Call stats for SNPs used by Yleaf to derive Y chromosome haplogroup for sample HU52. **Table S4f.** Call stats for SNPs used by Yleaf to derive Y chromosome haplogroup for sample HU53. **Table S4g.** Call stats for SNPs used by Yleaf to derive Y chromosome haplogroup for sample HU54. **Table S4f.** Call stats for SNPs used by Yleaf to derive Y chromosome haplogroup for sample HU55.

**S5 Table. List of SNPs used in Family Tree SNP collection based haplogroup derivation for ancient samples with read quality metrics. S5a Table.** List of SNPs used in Family Tree SNP collection based haplogroup derivation for HU3B and HU52 with read quality metrics. HU3B and HU52 are derived for the R-SUR51 haplogroup based on partial match (16 out of 23 SNPs ; bolded) with the SNPs defining this haplogroup. SNPs with ambiguous calls are highlighted in red. " ./" indicates ancestal allele; "NC" stands for no coverage at the given position; NA stands for indels that could not be assessed with this method. **S5b Table.**  List of SNPs used in Family Tree SNP collection based haplogroup derivation for HU3G with read quality metrics. HU3G is derived for J-ZS7626 haplogroup based on partial match (6 out of 25 SNPs; bolded) with with SNPs defining this haplogroup. SNPs with ambiguous calls are highlighted in red. " ./" indicates ancestral allele; "NC" stands for no coverage at the given position. **S5c Table.** List of SNPs used in Family Tree SNP collection based haplogroup derivation for HU4H with read quality metrics. HU4H is derived for haplogroup R-PF6658 and likely R-BY3642 based on complete match for the 3 SNPs defining these haplogroups (bolded). SNPs with ambiguous calls are highlighted in red. " ./" indicates ancestral allele; "NC" stands for no coverage at the given position. **S5d Table**. List of SNPs used in Family Tree SNP collection based haplogroup derivation for HU53 with read quality metrics. HU53 is derived for haplogroup E-BY4992 based on complete match for SNPs defining two consecutive haplogroups (E-BY4991 and E-BY4992) at positions covered (2/10; bolded). SNPs with ambiguous calls are highlighted in red. " ./" indicates ancestral allele; "NC" stands for no coverage at the given position. **S5e Table.** List of SNPs used in Family Tree SNP collection based haplogroup derivation for HU54 with read quality metrics. HU54 is derived for haplogroup R-Y2608 based on complete match for two consecutive haplogroups immediately upstream from R-YP1626 (R-2609, R-2608) at positions covered (4/5 ; bold). SNPs with ambiguous calls are highlighted in red. " ./" indicates ancestal allele; "NC" stands for no coverage at the given position; NA stands for indels that could not be assessed with this method. **S5f Table.** List of SNPs used in Family Tree SNP collection based haplogroup derivation for HU55 with read quality metrics. HU55 is derived for haplogroup (R-BY41605) based on an almost complete match for haplogroups R-BY11543 and R-BY41605 (for SNPs covered; 7/8 and 1/1, respectively; bold). SNPs with ambiguous calls are highlighted in red. " ./" indicates ancestal allele; "NC" stands for no coverage at the given position; NA stands for indels that could not be assessed with this method.

**S6 Table. Modern samples’ NGS data statistics and most derived identifiable haplogoups based on FamilyTree SNPs using FamilyTree nomenclature.**

**S7 Table. Derivation of the SUR51, ARP and UVD haplogroups. S7a Table.** Derivation of the ARP and UVD haplogroups. Shared and discrepant SNPs are shown for four individuals used in this analysis: Column H represents a modern day Bashkir individual from the Burzyansky region derived for all SNPs defining the SUR51 haplogroup; Column J represents the Árpád Dynasty member Béla III (HU3B); Column L represents the Árpád Dynasty member HU52; Column N represents the individual from modern day Serbia. SUR51 SNPs for which the Árpád Dynasty members and the individual from modern day Serbia carry the ancestral allele are bolded (7 total). SNPs with ambiguous calls highlighted in red. **S7b Table.** Derivation of the ARP and UVD haplogroups. Shared and discrepant SNPs are shown for four individuals used in this analysis: Column H represents a modern day Bashkir individual from the Burzyansky region who is ancestral for all SNPs defining the ARP haplogroup; Column J represents the Árpád Dynasty member Béla III (HU3B); Column L represents the Árpád Dynasty member HU52; Column N represents the individual from modern day Serbia. R-ARP SNPs for which the Árpád Dynasty members and the individual from modern day Serbia are derived for are bolded (9 total). **S7c Table**. Derivation of the UVD haplogroups. Shared and discrepant SNPs are shown for four individuals used in this analysis: Column H represents a modern day Bashkir individual from the Burzyansky region who is ancestral for all SNPs defining the UVD haplogroup; Column J represents the Árpád Dynasty member Béla III (HU3B); Column L represents the Árpád Dynasty member HU52; Column N represents the individual from modern day Serbia. R-UVD SNPs for which only the individual from modern day Serbia is derived for are bolded (9 total).

**S8 Table. SNPs used for Y Chromosome haplogroup derivation past R-Z2123**. All SNPs that fall outside the region recommended by Poznik et al. (13) for Y chromosome haplogroup derivation are highlighted in yellow. Only SNPs that fall within the recommended area were used for generation of the R-Z2123 phylogenetic tree in (Figure 3).
