# Supplementary figures and images for: Determination of the phylogenetic origins of the Árpád Dynasty based on Y chromosome sequencing of Béla the Third
Source: Eur J Hum Genet. 2020 Jul 7;29(1):164–72. doi: 10.1038/s41431-020-0683-z (PMC7809292; doi:10.1038/s41431-020-0683-z)

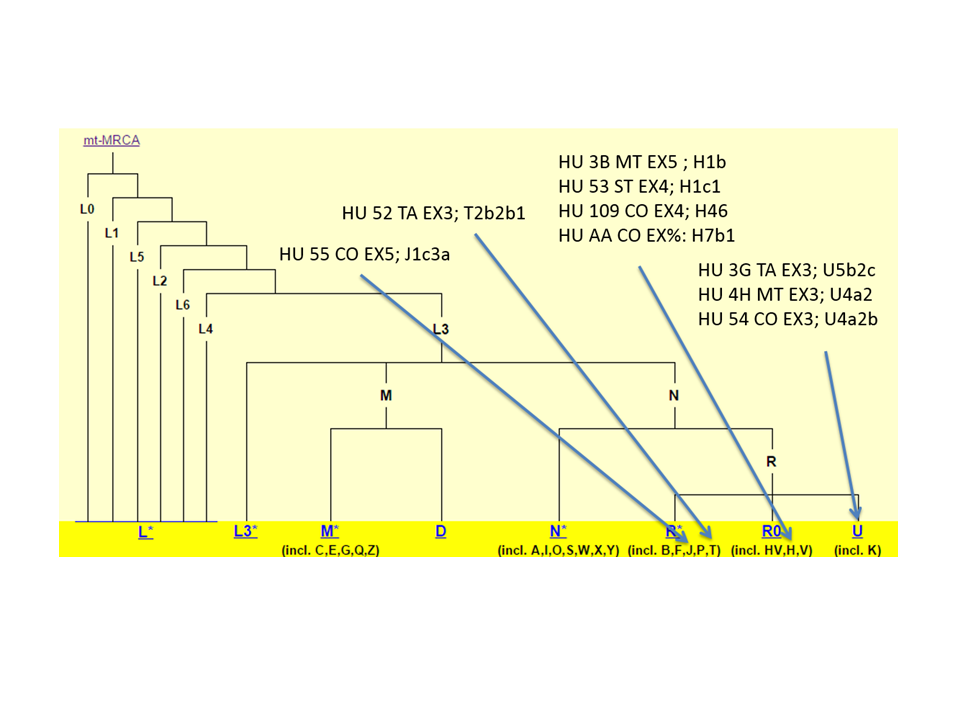

Supplement: Supplementary file 2 — S1 Figure [file 41431_2020_683_MOESM2_ESM.tif]

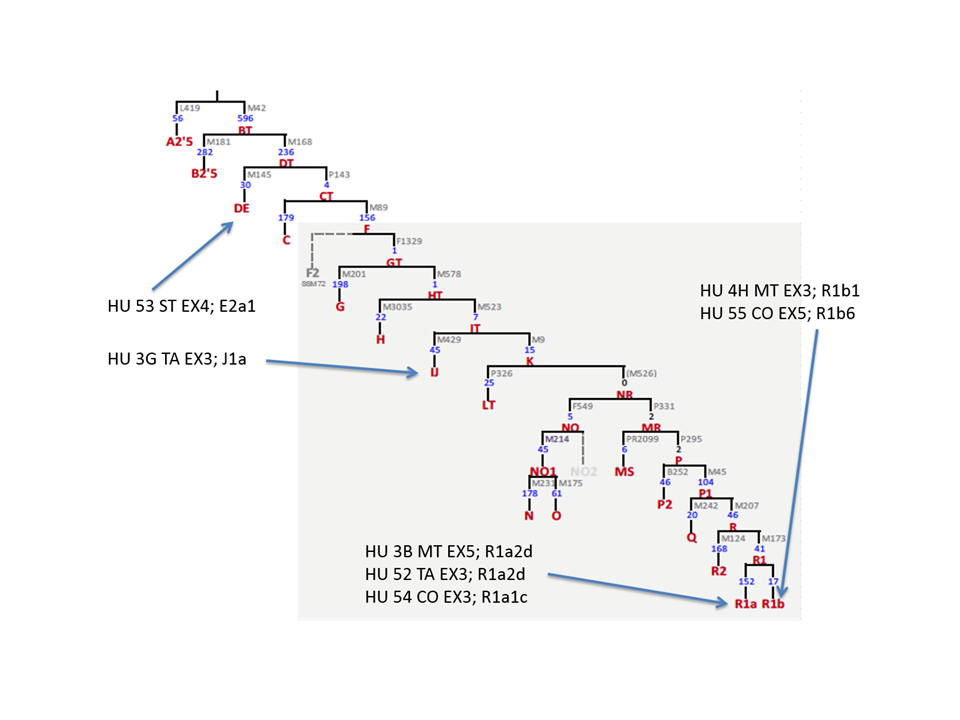

Supplement: Supplementary file 3 — S2 Figure [file 41431_2020_683_MOESM3_ESM.tif]
